# Seroprevalence of EV-A71 and EV-D68 antibodies in the paediatric and adolescent population, France, 2023 to 2025

**DOI:** 10.2807/1560-7917.ES.2026.31.44.2600054

**Published:** 2026-11-05

**Authors:** Hélène Chabrolles, Audrey Mirand, Benoît Visseaux, Marie Louchet-Ducoroy, Sonia Burrel, Mathilde Gay, Eline Brémond, Cyril Debuysschere, Mouna Lazrek, Marion Jeannoel, Isabelle Schuffenecker, Léa Luciani, Véronique Venard, Evelyne Schvoerer, Céline Bressollette-Bodin, Audrey Rodallec, Elyanne Gault, Laurent Andreoletti, Thibault Tromeur, Juliette Besombes, Marie Gueudin, Pauline Trémeaux, Sébastien Lhomme, Stéphanie Marque-Juillet, Céline Lambert, Maxime Bisseux, Jean-Luc Bailly, Cécile Henquell

**Affiliations:** 1 CHU Clermont-Ferrand, Service de Virologie, Centre National de Référence des Entérovirus et Parechovirus, Clermont-Ferrand, France; 2 Université Clermont Auvergne, UMR CNRS 6023-LMGE, Clermont-Ferrand, France; 3 Département d’infectiologie, Laboratoire Cerba, Cerba Healthcare, Frépillon, France; 4 CHU Amiens-Picardie, Service de Virologie, Amiens, France; 5 CHU Bordeaux, Service de Virologie, Bordeaux, France; 6 Université de Bordeaux, CNRS UMR 5234, Fundamental Microbiology and Pathogenicity, Bordeaux, France; 7 CHU Brest, Unité de Virologie, Brest, France; 8 CHU Lille, Service de Virologie, Lille, France; 9 Hospices Civils de Lyon, Département de Virologie, Centre National de Référence des Entérovirus et Parechovirus, laboratoire associé, Lyon, France; 10 Assistance Publique-Hôpitaux de Marseille, Service de Virologie Aiguë et Tropicale, Marseille, France; 11 CHU Nancy, Service de Virologie, Nancy, France; 12 Université de Lorraine, CNRS, LCPME, Nancy, France; 13 CHU Nantes, Service de Virologie, Nantes, France; 14 Assistance Publique-Hôpitaux de Paris, Hôpital Ambroise Paré, Service de Microbiologie, Boulogne-Billancourt, France; 15 CHU Reims, Service de Virologie, Reims, France; 16 Université de Reims Champagne-Ardenne, CardioVir UMR-S 1320, Reims, France; 17 CHU Reims, Service de Pédiatrie, Reims, France; 18 CHU Rennes, Service de Virologie, Rennes, France; 19 CHU Rouen, Service de Virologie, Rouen, France; 20 CHU Toulouse, Service de Virologie, Toulouse, France; 21 CH Versailles, Service de Biologie, Versailles, France; 22 CHU Clermont-Ferrand, DRCI, Unité de Biostatistiques, Clermont-Ferrand, France

**Keywords:** seroepidemiologic study, EV-A71, EV-D68, metropolitan France, neutralizing antibodies

## Abstract

**BACKGROUND:**

Enteroviruses (EV)-A71 and EV-D68 are potentially associated with severe clinical conditions, especially in infants and young children. Population susceptibility to these viruses is unknown in France.

**AIM:**

We sought to estimate seroprevalence of EV-A71 and EV-D68 neutralising antibodies (nAbs) among children and adolescents living in France.

**METHODS:**

A stratified sampling approach based on sex, age and geographical area of residence was applied to obtain sera for routine diagnostic from ˂ 18-year-olds in metropolitan France. A total of 501 serum samples collected between 2023 and 2025 from 0–17-year-olds were included in the investigation. Neutralising activity was tested against EV-A71 sub-genogroup C1 and EV-D68 subclade B3 strains isolated in 2016, allowing to determine seropositivity and nAb titre. Data were analysed overall, by sex, age group and geographical area.

**RESULTS:**

For EV-A71, the overall estimated seroprevalence was 43.7% (95% CI: 39.3−48.2) while for EV-D68 this reached 96.0% (95% CI: 93.9–97.5). No differences according to sex were observed. Unlike EV-D68, EV-A71 seropositivity rate varied significantly by age group (p = 0.002) and area of residence (p < 0.001). Notably, 23 of 25 children aged ˂ 1 year lacked EV-A71 protective antibodies. Concerning nAb titres, significant differences occurred across age groups both for EV-A71 (p = 0.004) and EV-D68 (p < 0.001). The nAb titres appeared as highest in 1 to under-2-year-olds for EV-A71 and 0 to under-1-year-olds for EV-D68.

**CONCLUSION:**

This seroprevalence study in France reveals a high proportion of newborns and infants susceptible to EV-A71 at potential risk of severe disease, contrasting with the high level of immunity against EV-D68 across all paediatric age groups.

**Key public health message**

**What did you want to address in this study and why?**

Enterovirus (EV)-A71 and EV-D68 can cause rare but serious illnesses that can include neurological complications, especially in children ˂ 5 years old. In France, the population immunity against EV-A71 and EV-D68 and age of primary exposure remain unknown. We aimed to estimate the immunity level (seroprevalence) to EV-A71 and EV-D68 in 0- to 17-year-olds in 2023–2025, overall, or according to age subgroups or geographical area of residence.

**What have we learnt from this study?**

Analysing serum samples of children and adolescents, we found that a very high proportion (96%) contained EV-D68 neutralising antibodies, reflecting a widespread virus circulation, while only 43.7% of serum samples had EV-A71 neutralising antibodies. Moreover, 23 of 25 sera from infants aged ˂ 1 year did not contain EV-A71 antibodies. Proportions of serum samples with EV-A71 antibodies also varied with children and adolescents’ area of residence.

**What are the implications of your findings for public health?**

Our study contributes to the national surveillance of EV infections and provides additional insights of epidemiological characteristics of EV-D68 and EV-A71. It defines vulnerable age groups lacking protective antibodies and geographical areas of low immunity for EV-A71. These findings can help target clinical surveillance efforts, anticipate healthcare needs and inform public health responses in the event of outbreaks.

## Introduction

Enteroviruses (EVs) are among the most common human viruses and cause a wide spectrum of highly contagious illnesses, with presentation ranging from subclinical or mild to severe, depending on host immunity and EV species (A–D) and type. While severe complications are rare, they can occur especially in newborns, children aged under 5 years, and immunocompromised individuals [1]. Among the over 116 EV types identified to date, EV-A71 and EV-D68 have emerged over the past two decades as major neurotropic pathogens.

Among EVs, EV-A71 is one of the main types associated with hand, foot and mouth disease (HFMD) and herpangina in preschool aged children. This virus has caused large-scale epidemics in the Asia-Pacific region since 2008 [2]. More importantly, it is the non-polio EV most frequently responsible for severe complications. These include myelitis and brain stem encephalitis, which can result in fatal neurogenic pulmonary oedema and heart failure [2]. Fatal outcomes due to EV-A71 have been reported during HFMD outbreaks, and in China the severity of EV-A71 infection has been found associated with young age (0–1 year) and living in rural areas [3]. These observations led to the development of EV-A71 vaccines in some Asian countries to reduce the occurrence of EV-A71-related neurological complications and mortality [4]. Outside the Asia-Pacific region, EV-A71 circulates at a low level, and in Europe and the United States (US) causes sporadic cases or small outbreaks every 2 or 3 years since the mid-2010s that can lead to severe neurological diseases [5-8].

Infection by EV-D68 primarily causes acute respiratory infections. Children under the age of 5 years, older adults (i.e. over 65 years of age), immunocompromised individuals and patients with underlying chronic conditions are at increased risk of developing severe respiratory disease requiring hospitalisation and potentially intensive care support [9]. Following its first identification in 1962, EV-D68 was initially only sporadically detected among cases of respiratory illnesses in the world; however, starting from 2009, increasing numbers of small outbreaks were reported across North America, Europe, and Asia [10]. In 2014, the first major outbreak of EV-D68 respiratory disease associated with acute flaccid myelitis, a polio-like paralysis with motor sequelae, was reported in the US [11] and subsequently in Europe [12,13]. Between 2014 and 2018, outbreaks occurred every 2 years, with cases of severe respiratory or neurological diseases [6,14-16]. In Europe, following the COVID-19 pandemic, the virus has re-emerged in many countries with yearly upsurges [17].

In France, the surveillance of EV infections has been organised since 1996 through the Enteroviruses Surveillance Network (RSE), coordinated by the National Public Health Agency (Santé publique France) and the National Reference Centre (NRC) for enteroviruses and parechoviruses, which has two laboratories in Clermont-Ferrand and Lyon. Around 40 hospital laboratories across metropolitan France transmit each month via an online form the number of hospitalised patients who test positive for EV, EV/rhinovirus, and parechovirus to the NRC. The laboratories also send positive samples to the NRC for molecular identification, along with associated patient demographic and clinical data. This system enables the NRC to provide detailed epidemiological information on circulating EV, rhinovirus, and parechoviruses types in metropolitan France associated with infections requiring hospitalisation, as well as insights into the clinical presentation and severity of disease.

However, many asymptomatic or mild EV infections are not captured by the national surveillance system. The presence of neutralising antibodies (nAbs) in serum is a widely accepted correlate of immunity and protection against severe disease associated with EV infection [1]. Therefore, seroepidemiologic surveys are essential to assess population susceptibility and to guide public health responses in the event of outbreaks. The objective of this study was to obtain recent estimates of the seroprevalence of EV-A71 and EV-D68 nAbs in children and adolescents in France, who constitute the main demographic group affected by EV infections.

## Methods

### Study design

We conducted a cross-sectional study to assess the seroprevalence of EV-A71 and EV-D68 nAbs in children and adolescents. A stratified sampling method based on age group, sex (male or female) and region of residence was used, proportionally according to demographic data from the French National Institute of Statistics and Economic Studies (INSEE) as of 1 January 2022. Seroprevalence data were then analysed in relation to the virological surveillance data, i.e. the number of EV infections reported to the NRC between 2014 and 2024.

### Sample collection

A total of 501 sera from individuals ˂ 18 years of age were consecutively selected from routine diagnosis specimens, excluding samples from immunocompromised children and adolescents. Of these, 496 were collected between March 2023 and February 2024. Five samples, all from infants under 1 year of age, were collected between March 2024 and May 2025, due to the limited availability of residual sera in this age group. All anonymised samples were stored at − 20 °C until analysis. They were grouped in five predefined age categories: < 1 year, 1 to < 2 years, 2 to < 5 years, 5 to < 10 years, and 10 to < 18 years, and covered the 13 administrative regions of metropolitan France. These, except for the Île-de-France region, were subsequently grouped into four broader geographical areas including:

-the northwest comprising the Bretagne, Normandie, Centre-Val-de-Loire, and Pays de la Loire;

-the northeast comprising Bourgogne-Franche-Comté, Grand Est and Hauts-de-France;

-the southwest comprising Nouvelle Aquitaine and Occitanie;

-and the southeast comprising Auvergne-Rhône-Alpes, Provence-Alpes-Côte d’Azur and Corse administrative regions.

### Cells and virus strains

Virus strains were isolated in 2016 from clinical samples in the NRC laboratory at the Clermont-Ferrand teaching hospital. The EV-A71 strain, identified by genotyping as EV-A71 sub-genogroup C1 (GenBank accession number: OQ091592), was propagated in rhabdomyosarcoma cells. The EV-D68 strain, identified as EV-D68 subclade B3 (GenBank accession number: MT791928), was propagated in A549 cells, an epithelial cell line derived from lung adenocarcinoma. The 50% tissue infective dose (TICD_50_) of each virus stocks was determined using an end-point dilution method previously described [18].

### Microneutralisation assays

All experiments were performed in a biosafety level 2 (BSL-2) laboratory. Serum aliquots were inactivated at + 56 °C for 30 min before testing. Duplicate twofold serial dilutions of serum were mixed in 96-well microplates with an equal volume of virus (100 TCID_50_) in a total volume of 100 µL, yielding final dilutions from 1:8 to 1:512. After incubation at + 37 °C under 5% CO_2_ for 1 h, 100 µL of medium containing cells were added. Plates were incubated at + 37 °C under 5% CO_2_ for 4 to 5 days. Cells were then checked by microscopic examination for cytopathic effect (CPE) indicating the presence of non-neutralised virus. Each plate included duplicate control: cell control, virus control (no serum) and serum control (no virus). A back-titration of virus infectivity was included in each run to confirm that 100 TCID_50_ has been used. Titres of nAbs were reported as the reciprocal of the highest serum dilution inhibiting ≥ 50% CPE in at least one of the two wells. Seropositivity was defined as a titre ≥ 8. Neutralisation titres < 8 were assigned a value of 4, and titres > 512 were assigned a value of 512.

To assess the specificity of the microneutralisation assay for the detection of anti-EV-A71 neutralising antibodies, two external controls were included: a commercially available human anti-EV-A71 VP1 antibody (H136), used as a positive control, and a commercially available human anti-EV-A71 VP4 antibody, used as a negative control. Similar specificity testing could not be performed for EV-D68 because no commercially available human EV-D68 antibodies were available.

### Virologic surveillance data

Across metropolitan France, approximately 40 hospital laboratories send samples which test positive for EV, EV/rhinovirus, and parechovirus to the NRC for molecular identification, along with associated patient demographic and clinical data. These include all EV positive samples from children aged < 1 year, immunocompromised patients, patients with meningitis or more severe neurological disease (e.g. myelitis, encephalitis, Guillain-Barré syndrome), patients with severe respiratory disease and any patient with atypical or severe presentation (e.g. sepsis in infants/young children, hepatitis, acute myocarditis, atypical eruption, chronic infection). To provide epidemiological context for observed nAb levels among the 501 routine diagnosis sera analysed in the study, we used the surveillance data from the NRC for enteroviruses and parechoviruses, collected through the RSE network. Because seroprevalence reflects cumulative exposure, we selected a 10-year window (2014–2024) to capture broad temporal patterns in EV circulation.

### Statistical analysis

The required sample size for the seroprevalence assessment of EV-A71 and EV-D68 nAbs in routine samples was calculated based on the expected proportion of the primary outcome and the desired margin of error. Assuming an expected EV infection prevalence of ca 50% (the most conservative estimate, requiring the largest sample size), the inclusion of 500 serum samples would yield an estimated proportion with a precision of ± 4.4%. Statistical analyses were performed using Stata software (version 16; StataCorp, College Station, TX, US). All tests were two-sided, with an alpha level set at 0.05. No adjustment for multiple comparisons was made in the secondary analyses, which were exploratory [19]. Categorical data were presented as counts and percentages, and quantitative data (age and nAb titres) as medians (25th; 75th percentiles). Seroprevalences of EV-A71 and EV-D68 were reported with 95% confidence intervals (95% CI), calculated using the binomial distribution. Factors associated with EV-A71 and EV-D68 infection (sex, age group, and geographical area of residence) were studied using the chi-square test or Fisher’s exact test, as appropriate. When the omnibus p value was < 0.05, post hoc multiple comparisons were conducted using the Marascuilo procedure. Multivariable analyses were performed using Firth’s penalised logistic regression, including sex, age group, and area of residence as covariates. Factors associated with nAb titres were studied using the Kruskal−Wallis test, followed, when appropriate (omnibus p value < 0.05), by Dunn’s post hoc multiple comparisons test. Due to limited sample sizes within some administrative French regions, analyses were performed considering five broader geographical areas (‘large areas’) of metropolitan France. More detailed results for each administrative region are available in Supplementary Tables.

## Results

### Demographic characteristics of patients and overall seroprevalences

The presence of EV-A71 and EV-D68 nAbs was assessed in 501 serum samples collected between March 2023 and May 2025 from children and adolescents aged 5 days to 17 years, of whom 51.1% (n = 256) were male and 48.9% (n = 245) female. Among children ˂ 1 year of age, median age was 7.1 months (25^th^; 75^th^ percentiles: 2.8; 9.3), with a range from 5 to 354 days. Demographic characteristics according to the large areas are presented in Table 1.

**Table 1:** Demographic characteristics of children and adolescents < 18 years old tested for neutralising antibodies against EV-A71 and EV-D68, France, 2023−2025 (n = 501 serum samples)

French region of residence	Average number of children and adolescents during study period	Children and adolescents included in the study					
		Number	%	M	F	Sex ratio (M/F)	Median age in years (25^th^; 75^th^ percentiles)
Île-de-France	2,809,259	102	20.4	52	50	1.04	9 (5; 15)
Northwest region	2,763,699	103	20.6	53	50	1.06	9 (5; 14]
Northeast region	3,032,454	107	21.4	54	53	1.02	10 (5; 15)
Southwest region	2,349,620	85	17.0	43	42	1.02	10 (5; 15)
Southeast region	2,859,823	104	20.8	54	50	1.08	9 (5; 15)
Total	13,814,855	501	100	256	245	1.04	10 (5; 15)^a^

EV: enterovirus; F: female; M: male.

^a^ This is not the total (as specified by the line heading), but the median age in years (25^th^; 75^th^ percentiles) of the children and adolescents included in the study.

Information on sex was collected as a binary variable (M/F) in each medical record.

At the threshold titre of eight, the overall estimated seroprevalence for EV-A71 and EV-D68 was 43.7% (95% CI: 39.3–48.2) and 96.0% (95% CI: 93.9–97.5), respectively (Table 2). Seroprevalence differed significantly across age groups for EV-A71 (p = 0.002), unlike for EV-D68 (p = 0.32). The proportion of samples positive for EV-A71 nAbs increased gradually with age from two of 25 in ˂ 1-year-olds to 115 of 235 (48.9%) in 10– ˂ 18-year-olds. In contrast, EV-D68 seroprevalence was remarkably high in all age groups, with all serum samples among children ˂ 2 years old being seropositive. The lowest proportion of EV-D68 seropositive children (92.9%) was observed in the 5– ˂ 10 years age group. Seropositivity rates for both EVs were not related to sex.

**Table 2:** Seropositivity rates for EV-A71 and EV-D68 nAbs in children and adolescents < 18 years old, France, 2023–2025 (n = 501 serum samples)

Variable	Number of sera tested	EV-A71 positive			p value	EV-D68 positive			p value
		Number	%	95% CI		Number	%	95% CI	
Overall	501	219	43.7	39.3–48.2	NA	481	96.0	93.9–97.5	NA
Sex									
Male	256	110	43.0	36.8–49.3	0.73	244	95.3	92.0–97.6	0.42
Female	245	109	44.5	38.2–51.0		237	96.7	93.7–98.6	
Age group, years									
0 to ˂ 1	25	2	NA^a^	1.0–26.0	0.002	25	NA^a^	86.3–100	0.32
1 to ˂ 2	25	9	NA^a^	18.0–57.5		25	NA^a^	86.3–100	
2 to ˂ 5	75	29	38.7*	27.6–50.6		73	97.3	90.7–99.7	
5 to ˂ 10	141	64	45.4*	37.0–54.0		131	92.9	87.3–96.5	
10 to ˂ 18	235	115	48.9*	42.4–55.5		227	96.6	93.4–98.5	

CI: confidence interval; EV: enterovirus; nAbs: neutralising antibodies; NA: not applicable.

Seropositivity rates were calculated using a threshold titre of eight.

^a^ Percentages are not presented as the proportions are based on small denominators; hence the numbers constituting the proportions (which are presented in the table) are considered more informative.

* Significant difference (p < 0.05) compared with the < 1-year-old age group (pairwise comparisons).

### Neutralising antibody titres and geometric mean titres according to age

Significant differences in median nAb titres were observed according to age groups for EV-A71 (p = 0.004) and EV-D68 (p < 0.001) (Table 3). For EV-A71 (Figure 1A), only two of 25 children under the age of 1 year were protected (Table 2). These two infants, aged 30 days and 7 months, had low nAb titres of 16 and 32, respectively. Among the nine children aged 1– < 2 years who were seropositive, seven had seroconverted with a high protective titre of ≥ 256. Of note, one child in this age group had a low titre of 32, very likely due to a recent natural infection given this patient’s age. For seropositive children, the EV-A71 nAb level appeared to be the highest in the age group of 1– < 2 years (geometric mean titre (GMT) = 256). It then seemed to decrease with age, with the GMT being 183 in 2– < 5-year and 5– < 10-year-olds, and 117 in the 10– < 18-year-olds (Figure 1A).

**Table 3:** Neutralising antibody titres against EV-A71 and EV-D68 in children and adolescents < 18 years old, France, 2023–2025 (n = 501 samples)

Variable	Number of sera tested	nAb titres^a^ median (25th; 75th percentiles)			
		EV-A71	p value	EV-D68	p value
Overall	501	4 (4; 128)	NA	256 (128; 512)	NA
Sex					
Male	256	4 (4; 128)	0.88	256 (128; 512)	0.24
Female	245	4 (4; 128)		256 (128; 512)	
Age group, years					
0 to ˂ 1	25	4 (4, 4)	0.004	512 (512; 512)	< 0.001
1 to ˂ 2	25	4 (4; 256)		128 (32; 256)*	
2 to ˂ 5	75	4 (4; 128)*		256 (64; 512)*	
5 to ˂ 10	141	4 (4; 256)*		256 (128; 512)*	
10 to ˂ 18	235	4 (4; 128)*		256 (128; 512)*	

EV: enterovirus; NA: not applicable; nAb: neutralising antibody.

^a^ Neutralisation titres below eight were assigned a value of four, and those above 512 were assigned a value of 512.

* Significant difference (p < 0.05) compared with the < 1-year-old age group (pairwise comparisons).

**Figure 1:**
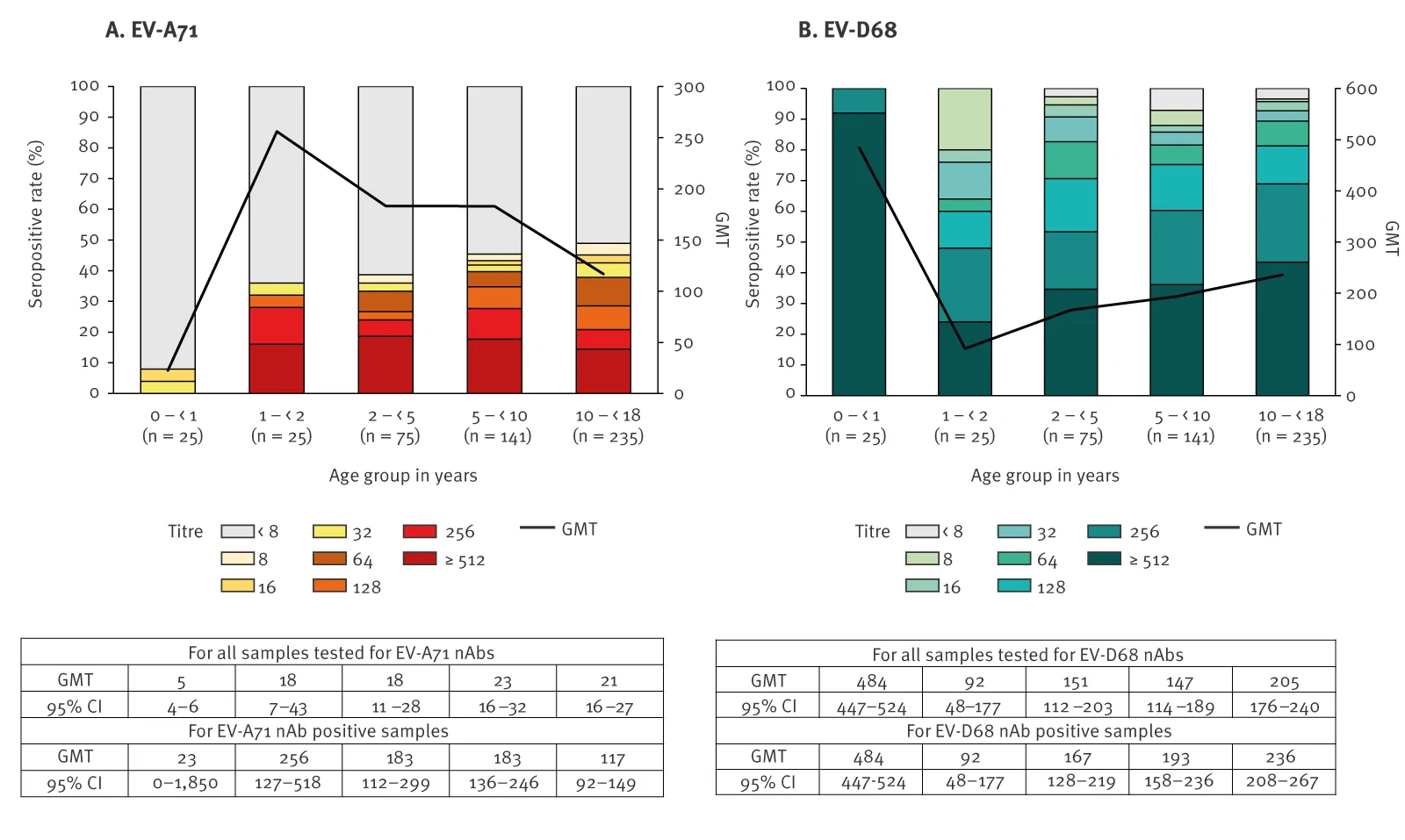
(A) EV-A71 and (B) EV-D68 seroprevalence and geometric mean neutralising antibody titre in children and adolescents < 18 years old, by age group, in metropolitan France, 2023–2025 (n = 501 samples)

CI: confidence interval; EV: enterovirus; GMT: geometric mean titre.

Samples were recorded seropositive if neutralisation was achieved at serum dilution of 1:8. Coloured bars indicate the percentage of samples displaying neutralising antibody titres < 8 to ≥ 512. The solid black line indicates the geometric mean titres calculated among seropositive serum samples.

In contrast, all neonates and infants under 1 year old were protected against EV-D68 (Figure 1B). In addition, all 25 had a high nAb titres, with two having a titre of 256 and 23 a titre ≥ 512. All 25 children of 1– ˂ 2 years old were also protected, despite half of them (n = 12) having nAb titres of 256 or ≥ 512. The proportion of children and adolescents protected with high titres (≥ 256) then re-increased significantly with age from 2 to < 18 years (p = 0.02). Consistently, the GMT in seropositive children and adolescents was the highest in the 0– ˂ 1-year-old group (GMT = 484). It dropped to 92 in the 1– ˂ 2-year-old group and then increased with age to reach 236 in the group of 10– ˂ 18-year-olds.

No sex-specific difference in EV-A71 or EV-D68 nAb titres was observed according to the age group (data not shown).

### Seroprevalence and neutralising antibody titres by large geographical area of residence

Seroprevalence of EV-A71 was significantly associated with large areas (p < 0.001), independently of children and adolescents’ age (adjusted p < 0.001). Seropositivity rates ranged from 32.7% in the northeast large region to 76.5% in Île-de-France (Figure 2A). Notably, the median nAb titres in Île-de-France were significantly higher than those observed in other regions (p < 0.001, data not shown). Consistent with nAb titres, the GMT of EV-A71 in positive serum samples was higher in Île-de-France than in other regions. More detailed information by administrative regions is presented in Supplementary Table S1.

**Figure 2:**
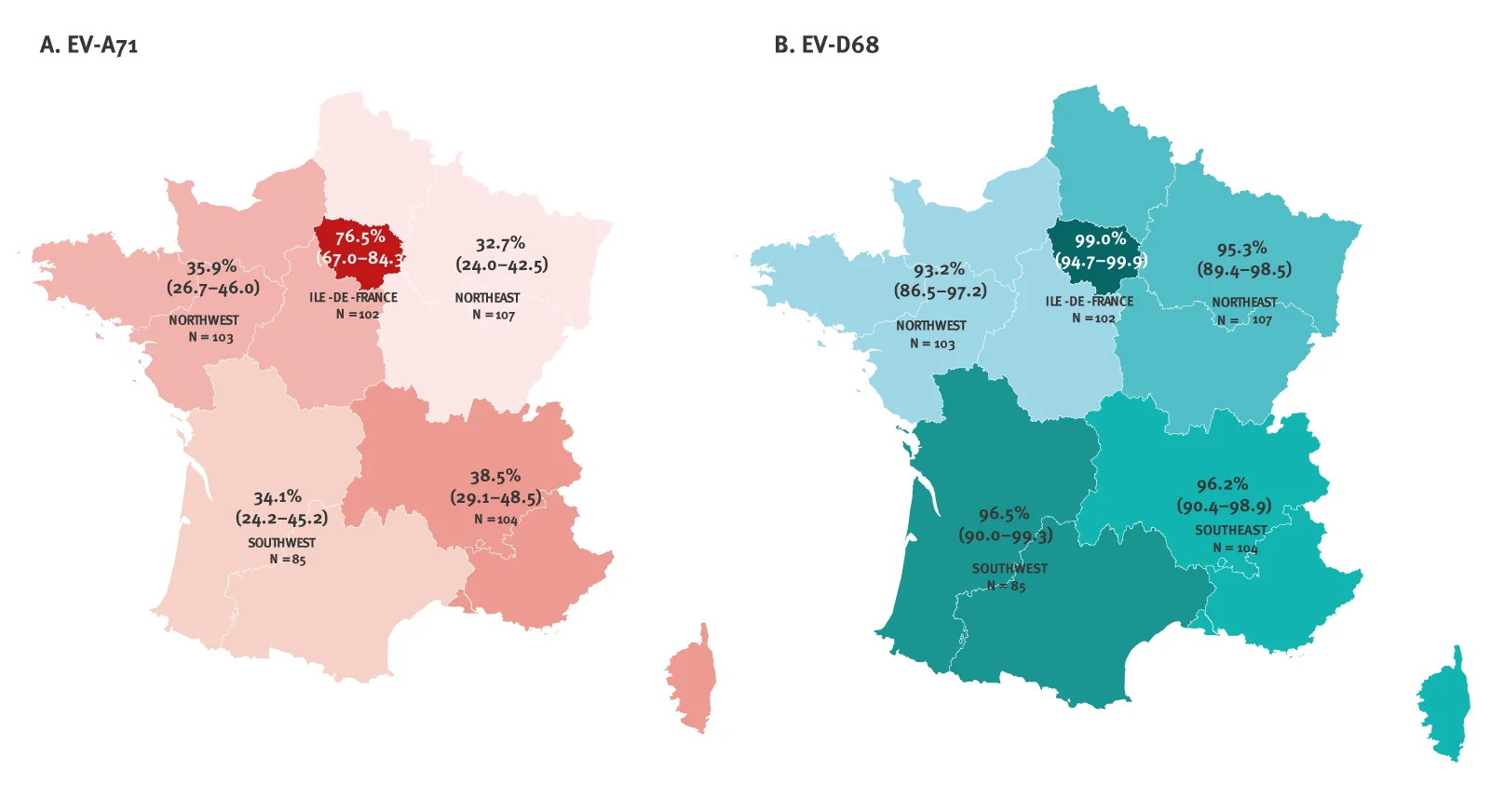
Seropositivity rates against (A) EV-A71 and (B) EV-D68 in children and adolescents < 18 years old, by large geographical region, France, 2023−2025 (n = 501 samples)

Source: Bing, GeoNames Microsoft TomTom.

EV: enterovirus.

Seropositivity rates are expressed as percentages (95% confidence interval). The numbers of serum samples (N) analysed per large geographical areas are indicated on the maps.

Seroprevalence of EV-D68 was not significantly associated with the large areas of residence (p = 0.32). Seropositivity rates ranged from 93.2% in northwest large area to 99.0% in Île-de-France region (Figure 2B). The nAb titres were not significantly associated with the large areas (p = 0.19, data not shown), and the GMTs were quite similar across areas. More detailed information by administrative regions is presented in Supplementary Table S2.

Finally, no sex-specific difference in EV-A71 or EV-D68 nAb titres was observed according to large areas (data not shown).

### Virologic surveillance of EV-A71 and EV-D68 infections in hospitalised patients

Between 2014 and 2024, a total of 25,475 EV infections were reported to the NRC by the RSE network. Of these, 76.9% (n = 19,578) EV-positive samples were referred to the NRC for genotyping. An EV type was successfully identified in 88.4% (17,309/19,578) of the samples. Among the EVs identified, 3.0% (522/17,309) and 10.7% (1,851/17,309) were EV-A71 and EV-D68, respectively. Their annual distribution showed biennial or triennial outbreak patterns before the COVID-19 pandemic, followed by disturbed dynamics from 2021 onwards (Figure 3).

**Figure 3:**
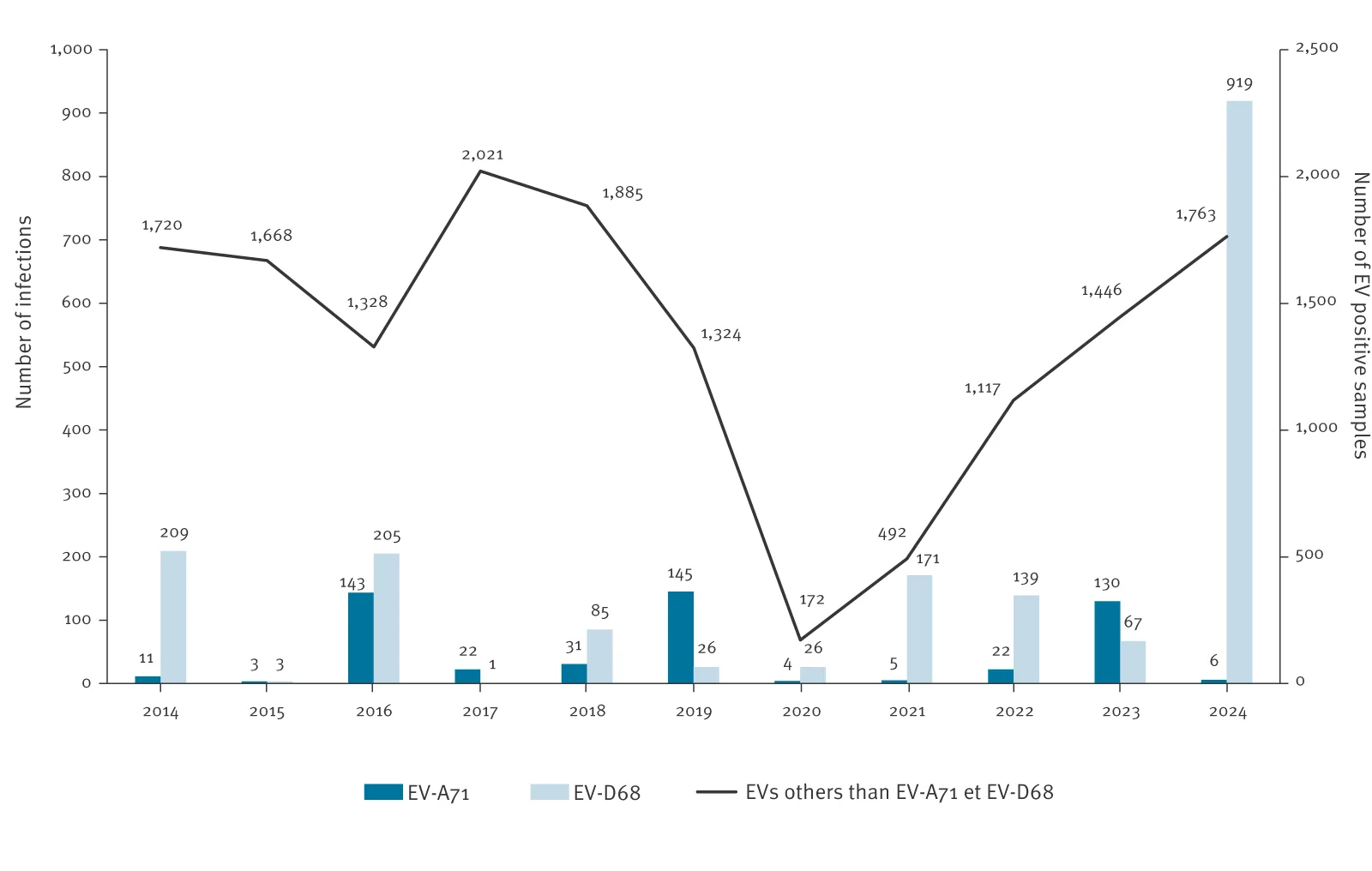
EV-A71 (n = 522) and EV-D68 (n = 1,851) infections identified from EV-positive samples referred to the National Reference Centre for enteroviruses and parechoviruses throughout France by the RSE network, 2014–2024 (n = 17,309 total samples)

EV: enterovirus; RSE: French national hospital laboratory network for the EV surveillance.

The solid black line indicates the number of infections with samples referred to the National Reference Centre, which were successfully genotyped.

Most EV-A71 and EV-D68 positive samples were obtained from hospitalised patients younger than 18 years old (409/522, 78.4% and 1,234/1,851, 66.7%, respectively). Among these patients, the main age group affected by EV-A71 and EV-D68 infections was the 0– < 1 year-group, with 57.9% (237/409) and 33.9% (418/1,234) of cases, respectively (Figure 4A and 4B). Overall, during the study period, 91.4% (374/409) of EV-A71 and 75.0% (926/1,234) of EV-D68 paediatric cases leading to hospitalisation were reported in children aged less than 5 years old.

**Figure 4:**
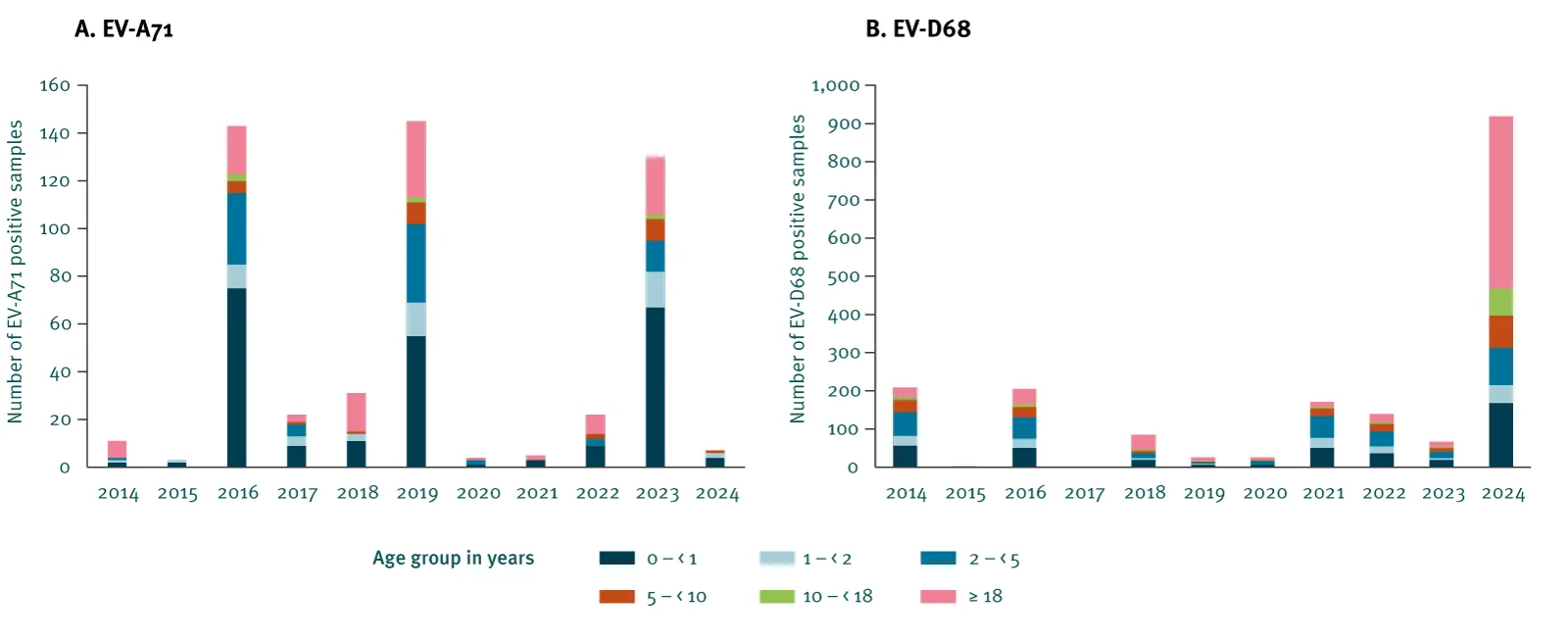
Age distribution of EV-A71 (A) and EV-D68 (B) infection cases referred to the National Reference Centre for enteroviruses and parechoviruses, France, 2014–2024 (n = 17,309)

EV-A71: enterovirus A71; EV-D68: enterovirus D68.

## Discussion

This study is the first seroepidemiologic investigation conducted in metropolitan France to assess population immunity against EV-A71 and EV-D68 infections in individuals aged 0– < 18 years. The overall seroprevalences of EV-A71 (43.7%) and EV-D68 (96.0%) nAbs differed substantially, suggesting that transmissibility, circulation dynamics, and pathogenic profiles differed between the two EV types.

Unlike most EVs, EV-D68 shares several characteristics with rhinoviruses and is predominantly transmitted via the respiratory route, resulting in easy and rapid spread in the general population. Surveillance data from the French NRC, consistent with other European reports [17,20], documented rapid EV-D68 resurgence in 2021 and sustained circulation thereafter. During the sampling period of this study, only a few cases were reported in 2023, preceding a marked increase in 2024 [21,22]. The increase in reported EV-D68 infections could be partially related to the widespread adoption of multiplex molecular assays detecting EV/rhinoviruses and the increased testing of respiratory specimens since the COVID-19 pandemic. Nevertheless, several serosurveys reviewed by Jorgensen et al. [23], found higher prevalence after 2014, suggesting that EV-D68 transmission has increased globally over time. All these serosurveys showed that seropositivity increased rapidly after the first year of life, when maternal Abs wane, and reached 100% in young adults [24-26]. In our study, the seroprevalence among children and adolescents living in France was particularly high (96.0%) and provided further evidence of extensive community circulation, with no significant difference between large geographical areas in the country. The highest EV-D68 nAb titres and GMT were observed in the 0– < 1-year group, suggesting the presence of maternal Abs and/or early natural infection in all children. The lowest GMT was measured in the age group 1– < 2 years, although all remained seroprotected. The gradual re-increase in GMT across older age groups, combined with the small proportion of susceptible children and adolescents (ranging from 2.5 to 7.1%), suggested continuous or recurrent exposure to EV-D68 infections in this population in recent years. The duration of protection after EV-D68 infection, and the roles of mucosal immunity and repeated exposures in boosting immunity against infection or disease are yet unknown. One study from Japan suggested that EV-D68 nAbs wane rapidly within 12 months post-infection [27].

Although all EV types can cause neurological disease, EV-A71 is the most neuropathogenic non-polio EV. In France, NRC surveillance showed that EV-A71 cases are rarely reported outside small outbreaks occurring every 2 or 3 years. During our sampling period, infections increased in 2023 but remained at low levels in 2024 and further data also indicated limited circulation in 2025 [21,22]. In this regard, the national HFMD surveillance, an outpatient, syndromic, and virological surveillance operated by the French Paediatric and Ambulatory Research in Infectious Diseases network since 2017 also indicated that EV-A71 detection was inconsistent in HFMD outbreaks in France [28]. This supports the notion of low-level circulation of this EV type in the country. Even in some Asian countries experiencing major epidemics, EV-A71 seroprevalence was lower than that of EV-D68. For example, in Guangdong, China, seropositivity reached 59.2% before a large HFMD outbreak in 2010 and 68.4% afterward [29]. In the United Kingdom, overall seroprevalence in sera collected in 2006, 2011 and 2017 was 74.0%, with rates of 60.7–69.0% in individuals aged 1–20 years [30]. In contrast, our results evidenced a lower seroprevalence in France in the 0– < 18-year-old population, where more than half of the individuals (56.3%) were susceptible to EV-A71 infection. Importantly, our results revealed a gap in protective maternal nAbs in newborns and infants under 1 year of age, who are the most vulnerable to severe EV-A71 disease. Longitudinal studies have shown that maternal EV-A71 Abs wane rapidly and disappear usually by 6 months of age, explaining the vulnerability of children under 1 year old [31,32]. In our study, only two of 25 infants aged 0– < 1 year had EV-A71 nAbs, with low titres likely reflecting residual maternal antibodies. This low seroprotection suggests limited circulation of EV-A71 among women of childbearing age during the study period. The first evidence of natural infection appeared in children aged 1– < 2 years (9/25 seropositive), who also exhibited the highest GMTs. Seropositivity then increased gradually across older age groups reaching 48.9% in 10– < 18-year-olds, indicating cumulative exposure with age. However, GMTs in infected children and adolescents declined with increasing age, suggesting that reinfection during childhood is uncommon. A longitudinal study in China showed that infected children with low initial nAb titres (< 128) were more likely to revert to seronegativity [3]. However, it is not known whether naturally infected individuals with low or undetectable nAbs remain susceptible to infection and severe disease, or whether they are protected by long-lasting immunity. Seroprevalence of EV-A71 in France varied depending on the region. Île-de-France showed significantly higher seroprevalence rate and GMTs than other regions. This may reflect the region’s large population size and high international mobility, potentially leading to regular introductions of EV-A71 strains followed by limited geographical spread [33].

This study has several limitations. Inferring maternal immunity from antibody patterns in infants under 1 year of age requires caution, given the current lack of seroprevalence data in adults, and particularly in pregnant women or women of childbearing age, in France. Moreover, seroprevalence of infants (0– < 1 year) may depend on whether there was an epidemic in the previous year. The cross-sectional design does not allow reliable estimation of reinfection dynamics, and kinetics of antibody titres across age groups should therefore be interpreted carefully. Longitudinal seroepidemiologic studies or repeated age-stratified cross-sectional surveys would provide insights into EV circulation and immune dynamics in France. Comparisons of seroprevalence rates across serosurveys are also limited by differences in study periods, age groups, and reporting methods. In addition, there is no standardised operating procedure for EV seroneutralisation assays. In our study, we defined the seropositivity threshold as a titre of eight or greater. Increasing the threshold from eight to 16 slightly changed the EV-A71 seropositivity rate (from 43.7% to 40.5%) and the EV-D68 seropositivity rate (from 96.0% to 92.8%). Although other lineages were also detected, we used strains EV-A71 sub genogroup C1 and EV-D68 subclade B3 as challenge viruses, as these have been the predominant lineages circulating in France since 2014 and during the study period [21,22]. Previous studies have shown strong cross-neutralisation among EV-A71 genogroups, due conserved antigenic sites [34]. Although antigenic sites and amino acid substitutions that could affect neutralisation epitopes are less well characterised for EV-D68, several studies likewise suggested cross-neutralisation across clades [23,25]. The high seroprevalence of EV-D68 raises suspicion of a possible cross-reactivity with other EVs, which would bias interpretation [35]. However, we tested the same serum samples against both EV-A71 and EV-D68 and observed two distinct nAbs profiles without evidence of cross-neutralisation, consistent with findings from other studies [36,37]. Furthermore, the lack of signal in the negative control and the expected positive response in the positive control provide additional evidence of assay specificity, supporting the reliability of the observed seroprevalence estimates.

## Conclusion

Our findings demonstrate widespread exposure to EV-D68 within individuals aged 0– < 18 years in France. In contrast, a high proportion of children, especially newborns and infants of less than 1 year, remained susceptible to EV-A71 and at risk of severe disease in case of large outbreaks. These results highlight the relevance of integrating serological data with clinical surveillance to refine our understanding of the circulation dynamics of neuropathogenic viruses and to identify the most vulnerable populations. They also underscore the strategic importance of seroprevalence data for epidemic preparedness, including anticipating of healthcare needs in young children, informing adjustments to clinical surveillance systems, and guiding vaccine-development priorities.

## Data Availability

All relevant data are included in the manuscript and its Supplementary material. Additional data supporting the findings of this study are available from the corresponding author upon reasonable request.

## Supplementary Data

129000 Supplement S1
