# Supplementary material for: Seroprevalence of EV-A71 and EV-D68 antibodies in the paediatric and adolescent population, France, 2023 to 2025
Source: Euro Surveill. 2026 Nov 5;31(44):2600054. doi: 10.2807/1560-7917.ES.2026.31.44.2600054 (PMC13743896; doi:10.2807/1560-7917.ES.2026.31.44.2600054)
Supplement: Supplementary file 1 [file 26-00054_HENQUELL_Supplement.pdf]

This supplementary material is hosted by Eurosurveillance as supporting information alongside the article "Seroprevalence of EV-A71 and EV-D68 antibodies in the paediatric and adolescent population, France, 2023 to 2025" on behalf of the authors who remain responsible for the accuracy and appropriateness of the content. The same standards for ethics, copyright, attributions and permissions as for the article apply. Eurosurveillance is not responsible for the maintenance of any links or email addresses provided therein.

Table S1. Seropositivity rate and geometric mean titres of neutralising antibodies against EV-A71 according to the French region of residence.

	All samples					Positive samples		
		Seropositivity rate		Geometric mean titer			Geometric mean titer	
**Region of residence**	**n**	**%**	**95% CI**	**GMT**	**95% CI**	**n**	**GMT**	**95% CI**
**Ile-de-France**	**102**	**76.5**	**67.0-84.3**	**104**	**70-155**	**78**	**285**	**227-357**
**Northwest region**	**103**	**35.9**	**26.7-46.0**	**13**	**9-18**	**37**	**98**	**68-143**
Bretagne	27	29.6	13.8-50.2	11	6-22	8	117	38-365
Centre-Val de Loire	18	33.3	13.3-59.0	11	5-25	6	91	33-247
Normandie	27	37.0	19.4-57.6	11	6-21	10	69	28-167
Pays de la Loire	31	41.9	24.5-60.9	17	9-33	13	121	66-222
**Northeast region**	**107**	**32.7**	**24.0-42.5**	**11**	**8-15**	**35**	**91**	**59-142**
Bourgogne-Franche-Comté	17	47.1	23.0-72.2	20	8-55	8	128	51-324
Grand Est	41	29.3	16.1-45.5	10	6-17	12	96	40-229
Hauts-de-France	49	30.6	18.3-45.4	10	6-15	15	74	35-154
**Southwest region**	**85**	**34.1**	**24.2-45.2**	**12**	**8-17**	**29**	**101**	**63-160**
Nouvelle Aquitaine	42	35.7	21.6-52.0	12	7-20	15	88	42-185
Occitanie	43	32.6	19.1-48.5	12	7-20	14	116	61-221
**Southeast region**	**104**	**38.5**	**29.1-48.5**	**14**	**10-20**	**40**	**104**	**65-165**
Auvergne-Rhône-Alpes	65	44.6	32.3-57.5	18	11-29	29	114	65-200
Corse	2	50.0	1.3-98.7	6	0-462	1	8	1-1
Provence-Alpes-Côte d'Azur	37	27.0	13.8-44.1	10	6-16	10	104	43-250

CI, confidence interval; GMT, geometric mean titer.

Table S2. Seropositivity rate and geometric mean titres of neutralising antibodies against EV-D68 according to the French region of residence.

	All samples					Positive samples		
		Seropositivity rate		Geometric mean titer			Geometric mean titer	
**Region of residence**	**n**	**%**	**95% CI**	**GMT**	**95% CI**	**n**	**GMT**	**95% CI**
**Ile-de-France**	**102**	**99.0**	**94.7-99.9**	**234**	**194-283**	**78**	**244**	**205-290**
**Northwest region**	**103**	**93.2**	**86.5-97.2**	**137**	**102-184**	**96**	**177**	**139-226**
Bretagne	27	92.6	75.7-99.1	104	54-200	25	135	75-243
Centre-Val de Loire	18	94.4	72.7-99.9	114	61-214	17	139	84-229
Normandie	27	100	87.2-100	269	203-358	27	269	203-358
Pays de la Loire	31	87.1	70.2-96.4	107	54-211	27	174	99-307
**Northeast region**	**107**	**95.3**	**89.4-98.5**	**179**	**138-233**	**102**	**216**	**174-269**
Bourgogne-Franche-Comté	17	82.4	56.6-96.2	123	47-325	14	256	139-470
Grand Est	41	100	91.4-100	265	189-372	41	265	189-372
Hauts-de-France	49	95.9	86.0-99.5	147	100-217	47	172	123-241
**Southwest region**	**85**	**96.5**	**90.0-99.3**	**207**	**158-271**	**82**	**239**	**191-299**
Nouvelle Aquitaine	42	97.6	87.4-99.9	244	174-342	41	269	204-356
Occitanie	43	95.3	84.2-99.4	177	115-271	41	213	149-304
**Southeast region**	**104**	**96.2**	**90.4-98.9**	**158**	**120-209**	**100**	**184**	**144-235**
Auvergne-Rhône-Alpes	65	93.8	85.0-98.3	149	102-216	61	188	137-259
Corse	2	100	15.8-100	181	2-14798	2	181	2-14798
Provence-Alpes-Côte d’Azur	37	100	90.5-100	176	114-271	37	176	114-271

CI, confidence interval; GMT, geometric mean titer.
